# Supplementary material for: SimSurvey: An R package for comparing the design and analysis of surveys by simulating spatially-correlated populations
Source: PLoS One. 2020 May 11;15(5):e0232822. doi: 10.1371/journal.pone.0232822 (PMC7213729; doi:10.1371/journal.pone.0232822)
Supplement: S1 Appendix — (DOCX) [file pone.0232822.s001.docx]

# S1 Appendix: Case study

The construction of the **SimSurvey** package was motivated by a need to assess the sampling design of trawl surveys conducted along the Newfoundland and Labrador shelf by Fisheries and Oceans Canada. As a tangible first step, we focused our efforts on emulating data from the survey of cod in NAFO Subdivision 3Ps since it is one of the most dynamic and heavily sampled stocks in the region. Function settings were iteratively modified until it was difficult to distinguish the simulated data from real survey data; these values were set as the defaults for the main functions in the package (see [**S2 Appendix**](#s2-appendix-parameterisation) for more details on how these defaults were chosen and for guidance on how to modify these settings to suit specific needs). Here we evaluate the efficacy of alternate sampling protocols by assessing deviation of stratified estimates of abundance [20] from the true abundance available to the survey. We also evaluate designed-based estimators recommended by Aanes and Vølstad [21] that account for cluster sampling of ages. Although assessing model-based analyses was an option, we focused on the design-based analyses for simplicity and because of its widespread use. The code below displays the core defaults and will replicate the results of this case study. The last few lines of code will also replicate a simulation configured to test an alternate age sampling and analysis approach. Results produced here are the same as those produced and displayed in a series of figures in the [**test_surveys**](#test_surveys) and [**Interpretation**](#interpretation) sections of the paper.

# install.packages("remotes")
# remotes::install_github("PaulRegular/SimSurvey")
# library(SimSurvey)

set.seed(438)

abundance <- sim_abundance(
 ages = 1:20,
 years = 1:20,
 R = sim_R(log_mean = log(30000000),
 log_sd = 0.5),
 Z = sim_Z(log_mean = log(0.5),
 log_sd = 0.2,
 phi_age = 0.9,
 phi_year = 0.5),
 growth = sim_vonB(Linf = 120,
 L0 = 5,
 K = 0.1,
 log_sd = 0.1,
 length_group = 3)
)

distribution <- sim_distribution(
 abundance,
 grid = make_grid(x_range = c(-140, 140),
 y_range = c(-140, 140),
 res = c(3.5, 3.5),
 shelf_depth = 200,
 shelf_width = 100,
 depth_range = c(0, 1000),
 n_div = 1,
 strat_breaks = seq(0, 1000, by = 40),
 strat_splits = 2),
 ays_covar = sim_ays_covar(sd = 2.8,
 range = 300,
 phi_age = 0.5,
 phi_year = 0.9,
 group_ages = 5:20),
 depth_par = sim_parabola(mu = 200,
 sigma = 70)
)

surveys <- test_surveys(
 distribution,
 surveys = expand_surveys(set_den = c(0.5, 1, 2, 5, 10) / 1000,
 lengths_cap = c(5, 10, 20, 50, 100, 500, 1000),
 ages_cap = c(2, 5, 10, 20, 50)),
 n_sims = 5, n_loops = 200, cores = 3,
 q = sim_logistic(k = 2, x0 = 3)
)

alt_surveys <- test_surveys(
 distribution,
 surveys = expand_surveys(set_den = 2 / 1000,
 lengths_cap = c(5, 10, 20, 50, 100, 500, 1000),
 ages_cap = c(1, 2, 3, 5, 10)),
 n_sims = 5, n_loops = 200, cores = 3,
 q = sim_logistic(k = 2, x0 = 3),
 age_length_group = 3,
 age_space_group = "set",
 alk_scale = "set"
)

## Results

The default settings of sim_abundance and sim_distribution produces a dynamic population with a patchy and age-clustered distribution (Figure 1a, 3a). In general, the function defaults dictate that all ages tend to aggregate in fairly dense clusters and that low-density zones are relatively wide-spread. Surveys, therefore, tend to be characterized by regions with large catches interspersed with regions of no catch (Figure 4). The samples within each successful set are also variable and rarely include all length or age groups. This is because only moderate correlation ($\varphi_{\xi,\mathrm{age}}$ of 0.5) was imposed on the distributions of ages 1-5+, allowing individuals of different ages to occupy different locations, as is typically observed in the actual survey. These settings were chosen to simulated data that roughly correspond to actual survey data of cod from NAFO Subdivision 3Ps, where each age group between 1-4 tend to be caught at different locations while cod older than 4 years tend to be found at similar locations. In short, simulated data roughly emulate actual survey data with clustered catches and length samples with clear intra-haul correlation (Figure S1).

Figure S1: Comparison of a) real and b) simulated survey data where, from top to bottom, histograms of numbers caught across sets, distributions of set catches, and length-frequency distributions from three random sets are shown from a randomly selected year from the real and simulated 3Ps cod survey data. The colors of the length-frequency plots correspond to the colored locations. The deeper shades in the length-frequency plots represent numbers of fish sampled for age determination.

Using the test_surveys function, a series of surveys were run over the simulated population and stratified analyses were run on the data obtained. The stratified analyses provided estimates of total abundance, abundance at length and abundance at age. Different stages of sampling affect these estimates, specifically, set density affects all estimates, length sampling affects estimates of abundance at length and abundance at age, and age sampling affects abundance at age estimates.

The effect of set density on estimates of total abundance, abundance at length and abundance at age are clear across all figures generated from the test_surveys results (Figures 5, 6, 7, 8, 9). Across all fan plots, it is clear that the probability envelopes tighten (i.e. estimates are more precise) as set density is increased (Figures 5, 6, 7). Clear declines in RMSE are also apparent in estimates of abundance at length as set density is increased (Figure 8) and there are notable differences in the scale of RMSE in estimates of abundance at age as set density is increased (Figure 9).

Compared to increases in set density, the effects of increased length sampling effort on abundance at length estimates are less clear. Regarding estimates of abundance at length, RMSE appears to reach a plateau at when length sampling effort is increased to 100 measurements per set (Figure 8). Increasing the sampling rule above 100 measurements per set results in many more fish being measured, however, the increase in sampling effort is not matched with substantive declines in RMSE (Figure 8). Further, it appears that measuring fewer total fish at more locations is more beneficial than measuring many fish at fewer locations (Figure 8).

All levels of sampling affect the abundance at age estimates as set catches define the magnitude of the population, length sampling define the length distribution and data from the length-stratified age sampling is used to construct an age-length-key to convert length frequencies to age frequencies. Like the abundance at length estimates, the greatest improvements to RMSE come from increasing set density rather than sub-sampling effort (Figure 9). Specifically, decreasing the age sampling protocol below 10 ages sampled per length group per division appears to result in relatively small increases to RMSE (Figure 9). Length sampling effort, in contrast, has an uneven impact depending on the set density scenario. At low set densities ($D_{\mathrm{sets}}$ = 0.0005 sets / km^2^), RMSE declines when length sampling effort is increased from around 5 to around 100 measurements per set, and RMSE starts to increase as length sampling effort is increased beyond ~100 measurements per set; in fact, RMSE values appear higher at the highest length sampling scenario (1000 measurements / set) than the lowest (5 measurements / set; Figure 9). A similar pattern is apparent under the medium set density scenario ($D_{\mathrm{sets}}$ = 0.002 sets / km^2^), however, RMSE values under the lowest and highest length sampling scenarios are of similar magnitude. Finally, RMSE continues to decline with increased length sampling effort under the high set density scenarios ($D_{\mathrm{sets}}$ = 0.01 sets / km^2^; Figure 9).

Bias in stratified estimates of abundance at age is minimal when length-stratified age sampling is conducted and age-length keys are applied on a set-by-set basis (Figure 10a). Under this scenario, there also appears to be a plateau, rather than a valley, in RMSE values as length and age sampling effort is increased (Figure 10b). Like the default approach (division-wide age sampling and age-length key), RMSE declines when length sampling effort is increased from around 5 to 100 measurement per set, however, beyond 100 there is little change in the RMSE values (Figure 10b). In contrast to the length sampling axis, there is relatively little change in the age sampling axis; a small decline in RMSE is apparent as age sampling per length group per set is decreased from 1 to 3, with little change above this level of effort (Figure 10b).

## Discussion

The simple case study presented here revealed some expected, but also a few unexpected, patterns. First, and not surprisingly, there were clear improvements to precision in all population estimates as the number of sets were increased [41]. Second, stratified estimates of abundance at age were often biased and, in some cases, estimates were poorer when sub-sampling effort was increased. This result was unexpected because design-based estimators, such as the stratified analysis applied here, are expected to be unbiased [39] and large increases to sub-sampling effort could be expected to be relatively ineffective, but not detrimental. Results from stratified estimates of abundance at length, in contrast, better align with these expectations. By deduction, these results indicate that the issue stems from the intervening age-length key and not the design-based estimator.

Using an age-length key in conjunction with the length distribution to estimate abundance at age is standard procedure in the analysis of fisheries-independent survey data as only a small fraction of the catch are typically aged. The aging procedure is costly and time-consuming whereas length measurements are relatively easy to obtain. Ages are generally determined from length-stratified sub-samples of the catch and raw proportions of age-at-length are used to assign ages to fish in specific length groups. Age sampling is often spread across large spatial scales and age-length-keys are usually applied at this scale (i.e. age sampling may not occur at every set). Here we construct one age-length key for the division, as is done for the analysis of 3Ps cod. There is, however, a potential cost to the spatial scale of the key. Namely, it is unlikely that one age-length key is representative for the whole region because the probability of being a specific age given length varies in space [38]. This would not be an issue if there was no size (age) specific clustering, however, this may not always be the case because different size groups often occur in different places because of ontogenetic habitat shifts [42,43]. For instance, it is not uncommon for populations to form distinct nursery and spawning areas [44,45]. Because different age groups sometimes occur in different places, the translation of lengths to ages may be biased by the samples used to generate the age-length key. This bias is perhaps compounded by the length-stratified sampling of ages such that the ages sampled may be skewed towards sets with the most catch, especially under scenarios where length sampling effort is high. If this is the case, then the representativeness of the age-length key to the whole population could be diminished by excessive length sampling. In short, the primary cause of the problem is that the classic age-length key method assumes that fish are drawn from a simple random sample; this assumption, however, is rarely satisfied in practice [21].

While there are potential model-based solutions to bias introduced by large scale age-length keys (e.g. spatial age-length keys [38]), a design-based solution is to utilize estimators that account for multi-stage cluster sampling designs [21]. This approach was tested here and, indeed, it appears to resolve the bias in the stratified estimates of abundance at age. This implies that inference from the actual survey of 3Ps cod can be improved by a simple adjustment of the sampling design and analytical approach. In the context of stock assessment, and unbiased estimator is certainly the preferred option because under-estimates may lead to unnecessary cuts to a fishery and over-estimates may contribute to ill-advised quota increases and potentially avoidable population declines.

While much work has yet to be done, results from the simple simulation echo the growing body of literature which concludes that extra sub-sampling is an ineffective means of improving estimates relative to sampling more locations [10–12,34–36]. In general, the most significant source of variation in fisheries-independent surveys stems from set-to-set variation, and not variability from individual sub-samples. This is largely because fish caught together tend to be more similar than those in the general population [10]. Such cluster sampling can also introduce bias if the hierarchical sampling design is not explicitly accounted for [21]. Therefore, if the goal of a trawl survey is to maximize information, it is likely better to stop collecting correlated samples and, instead, focus efforts the next set and/or other species. This is simply one example of the questions that can be explored using **SimSurvey**.

**References**

41. Sutherland WJ. Ecological census techniques: A handbook. Cambridge University Press; 2006.

42. Dahlgren CP, Eggleston DB. Ecological processes underlying ontogenetic habitat shifts in a coral reef fish. Ecology. 2000;81: 2227–2240.

43. Galaiduk R, Radford BT, Saunders BJ, Newman SJ, Harvey ES. Characterizing ontogenetic habitat shifts in marine fishes: Advancing nascent methods for marine spatial management. Ecological Applications. 2017;27: 1776–1788.

44. Marteinsdottir G, Gudmundsdottir A, Thorsteinsson V, Stefansson G. Spatial variation in abundance, size composition and viable egg production of spawning cod (*Gadus morhua* L.) in icelandic waters. ICES Journal of Marine Science. 2000;57: 824–830.

45. Booth AJ. Incorporating the spatial component of fisheries data into stock assessment models. ICES Journal of Marine Science. 2000;57: 858–865.
