# Supplementary material for: SimSurvey: An R package for comparing the design and analysis of surveys by simulating spatially-correlated populations
Source: PLoS One. 2020 May 11;15(5):e0232822. doi: 10.1371/journal.pone.0232822 (PMC7213729; doi:10.1371/journal.pone.0232822)
Supplement: S2 Appendix — (DOCX) [file pone.0232822.s002.docx]

# S2 Appendix: Parameterisation

The default parameter values used in this package were chosen to emulate survey data from a specific cod population (see [**S1 Appendix**](#s1-appendix-case-study) for details) and, as such, these values ought to provide a reasonable place to start for another population of cod or similar groundfish. In this section we outline a series of recommended steps to take in order to tailor the default settings to suit other systems. Throughout this outline, we also provide some context on how default parameter values were chosen as this information may help guide a users’ choices.

## 1. Define survey area and protocol

Parameterising the survey design should be the easiest place to start as the area and protocol should be clearly defined. The survey area can be defined using make_grid or it can be manually constructed. For the case study, make_grid arguments were modified to create a grid with similar dimensions to the stratified design actually applied to the case study area (i.e. total area, shelf depth, number of strata, etc.). Also note that the resolution (res argument) of the grid was defined to be similar to the resolution at which sets are allocated in the survey. A square grid was chosen in lieu of the actual survey grid to create an area that avoids potential issues associated with land as a barrier while being sufficiently complex to test survey designs. When setting up a grid, be careful not to drastically increase the dimensions (x_range or y_range arguments) or resolution (res argument) as simulating the spatial noise over a large field can be computationally demanding.

With a survey grid defined, the settings of sim_survey can be modified to match the protocol of a users’ survey of interest. For the case study, the trawl dimensions (trawl_dim) were modified to match the dimensions of a standard trawl conducted in the region. Likewise, area is used to define the number of sets to allocate to a strata and the value used by default for set_den matches the protocol, as does the min_sets setting (i.e. at least two sets are conducted in each strata to facilitate stratified analyses). Finally, length and age sub-sampling settings were modified to match the protocol for the survey of cod in the case study area. The most difficult parameter to define in the sim_survey function is q. A logistic curve is used by default to define these unknown values and the sim_logistic parameters were tweaked to approximate the catchability curve supplied to the survey-based model that was used to assess the case study population [46]. A similar tactic can be applied to other cases with estimates of survey catchability.

## 2. Define abundance and growth parameters

Abundance and growth parameters are more difficult to define than the survey protocol as the true values are unknown. Nevertheless, spatially-aggregated age-structured models are frequently applied to assess fish populations and estimates from such models can be used to define sim_abundance argument settings. Alternatively, key values from these models (e.g. Z and N matrices) can be used instead of simulated values. Default parameters used in sim_abundance were largely inspired by estimates from the survey-based model used to assess the cod stock the case study was based on [46]. Based on this work, average total mortality was roughly centered around 0.5 ($\mu_{Z}$; argument log_mean in sim_Z closure) and we allowed it to make similar changes in magnitude through time and across ages by setting the standard deviation of log total mortality to 0.2 ($\sigma_{Z}$; argument log_sd in sim_Z closure) and the correlations to 0.9 and 0.5 in the age and year dimensions ($\varphi_{\delta,\mathrm{age}}$ and $\varphi_{\delta,\mathrm{year}}$; phi_age and phi_year), respectively. Likewise, sim_R values were modified to generate similar patterns in recruitment as those estimated by the model. We simulated data for ages 1 to 20 ($a$; ages argument) to capture the tails of the population, and 20 years were simulated ($y$; years argument) to capture some dynamics. Finally, von Bertalanffy growth parameters were roughly based on estimates from cod in the region [47]. While the choices made are obviously imperfect and ad hoc, it is not necessary to generate a perfect representation of the population as the goal here is to generate a dynamic population to survey. What is key is that a known population is simulated from which the performance of specific survey designs and analyses can be evaluated.

## 3. Define spatial parameters

Ideally this step would be no more difficult than step 2 as the inputs required could come from a spatially-explicit age-structured model fit to real data. Such estimates, however, are rare and we can confirm that no such model has been developed and fit to data from our case study population. Unless a user has access to such estimates, parameter estimates will need to be manually defined using visual comparisons of real and simulated data. A user will have an initial simulation of some survey data by following steps 1 and 2, and the plotting functions provided with the package will provide a means of visually exploring these data. For comparison purposes, actual survey data will need to be processed and visual patterns in the spatial distribution of each age will need to be inspected. It was in this step that we noticed that ages 1 to 4 tend to occupy different areas while ages 5+ occupy similar space for the cod population we focused on. Observing these patterns in real data motivated the coupling of the spatial noise of ages 5+ (group_ages argument in the sim_ays_covar closure), which forces this component of the population to occupy the same space, and the moderate value of 0.5 for the correlation in the spatial distribution across ages ($\varphi_{\xi,\mathrm{age}}$; phi_age argument in the sim_ays_covar closure). Year-to-year plots of the real data also revealed considerable inertia in the space occupied by each age group; this observation motivated a fairly high value of 0.9 for the correlation in the spatial distribution across years ($\varphi_{\xi,\mathrm{year}}$; phi_year argument in the sim_ays_covar closure). Finally, the range of the spatial correlation ($r$; range argument in the sim_ays_covar closure) was iteratively modified until the simulated population exhibited a similar level of patchiness as the real data, and the standard deviation of the age-year-space process ($\sigma_{\xi}$; sd argument in the sim_ays_covar closure) was increased to generate set catches with tails similar to those observed in the real data (cod catches in the area tend to be zero-inflated and heavy-tailed). Finally, visual inspection of the real data revealed that the largest catches tended to be centered on sets conducted in 200 m of water; a parabolic relationship, defined using the sim_parabola closure, with a mean of 200 m and standard deviation of 70 m approximated this pattern.

**References**

46. Ings D, Rideout R, Rogers R, Healey B, Morgan M, Robertson G, et al. Assessing the status of the cod (*Gadus morhua*) stock in NAFO Subdivision 3Ps in 2018. DFO Can Sci Advis Sec Res Doc. 2019;2019/069: v + 75p.

47. Cadigan N, Konrad C. A cohort time-series Von Bertalanffy growth model for Northern cod (*Gadus morhua*), and estimation of the age of tagged cod. DFO Can Sci Advis Sec Res Doc. 2016;2016/017: v + 37p.
