# Supplementary material for: SimSurvey: An R package for comparing the design and analysis of surveys by simulating spatially-correlated populations
Source: PLoS One. 2020 May 11;15(5):e0232822. doi: 10.1371/journal.pone.0232822 (PMC7213729; doi:10.1371/journal.pone.0232822)
Supplement: S3 Appendix — (DOCX) [file pone.0232822.s003.docx]

# S3 Appendix: Age-year-space covariance

The simulation applied in this paper was set-up to control covariance across ages, years and space. To do this we used a combination of Matérn covariance, to control the level of spatial aggregation, and the age-year covariance described in Cadigan [16], to control the level of similarity in distributions across ages and years. As described in Appendix A in Cadigan [16], the age-year covariance can be broken down into a series of AR(1) processes. We integrate Matérn covariance into this series of equations:

$$\xi_{a,y,s}\sim\left\{ \begin{matrix} MVN\left( 0,\frac{\sigma_{\xi}^{2}}{(1-\varphi_{\mathrm{age}}^{2})(1-\varphi_{\mathrm{year}}^{2})}\mathbf{R}_{s} \right) & a=1,y=1 & \\ MVN\left( \varphi_{\mathrm{year}}\xi_{1,y-1,s},\frac{\sigma_{\xi}^{2}}{(1-\varphi_{\mathrm{age}}^{2})}\mathbf{R}_{s} \right) & a=1,y>1 & \\ MVN\left( \varphi_{\mathrm{age}}\xi_{a-1,1,s},\frac{\sigma_{\xi}^{2}}{(1-\varphi_{\mathrm{year}}^{2})}\mathbf{R}_{s} \right) & a>1,y=1 & \\ MVN\left( \varphi_{\mathrm{year}}\xi_{a,y-1,s}+\varphi_{\mathrm{age}}(\xi_{a-1,y,s}-\varphi_{\mathrm{year}}\xi_{a-1,y-1,s}),\sigma_{\xi}^{2}\mathbf{R}_{s} \right) & a>1,y>1 & \end{matrix} \right.$$

Where $MVN$ indicates the multivariate normal distribution, $\sigma_{\xi}^{2}$ controls the variance of the process, $\varphi_{\xi,\mathrm{age}}$ and $\varphi_{\xi,\mathrm{year}}$ control correlation in the age and year dimension and $\mathbf{R}_{s}$ is defined by Matérn correlation:

$$\mathbf{R}_{s}=\frac{2^{1-\lambda}}{\Gamma(\lambda)}(\kappa\left| s_{i}-s_{j} \right|)K_{\lambda}(\kappa\left| s_{i}-s_{j} \right|)$$

where $\left| s_{i}-s_{j} \right|$ is the Euclidean distance between two locations, $\Gamma$ is the gamma function, $K_{\lambda}$ denotes the modified Bessel function of the second kind, and $\lambda$ and $\kappa$ control the smoothness and scale of the spatial process [18]. With this structure, simulated error is correlated across ages, years and space.
