# Supplementary material for: SimSurvey: An R package for comparing the design and analysis of surveys by simulating spatially-correlated populations
Source: PLoS One. 2020 May 11;15(5):e0232822. doi: 10.1371/journal.pone.0232822 (PMC7213729; doi:10.1371/journal.pone.0232822)
Supplement: S4 Appendix — (DOCX) [file pone.0232822.s004.docx]

# S4 Appendix: Stratified analysis equations

Standard notation for use in the analysis of stratified-random survey data (modified from [20]):

| **Equation** | **Description** |
| --- | --- |
| $H$ | Number of strata ($h=1,2,.....,H$) |
| $A_{h}$ | Area of the $h^{th}$ stratum |
| $A_{\mathrm{trawl}}$ | Area covered by a standard trawl |
| $N_{h}=\frac{A_{h}}{A_{\mathrm{trawl}}}$ | Total number of sample units in the $h^{th}$ stratum |
| $n_{h}$ | Total number of units sampled in the $h^{th}$ stratum ($i=1,2,.....,n_{h}$) |
| $N=\sum_{h=1}^{H} N_{h}$ | Total number of sample units in the survey |
| $n=\sum_{h=1}^{H} n_{h}$ | Total number of observations in the survey |
| $W_{h}=\frac{N_{h}}{N}$ | Stratum weight |
| $f_{h}=\frac{n_{h}}{N_{h}}$ | Sampling fraction in the $h^{th}$ stratum |
| $\overline{I}_{h}=\sum_{i=1}^{n_{h}} \frac{I_{h,i}}{n_{h}}$ | Sample mean in the $h^{th}$ stratum, where $I_{h,i}$ is the $i^{th}$ observation in the $h^{th}$ stratum |
| $s_{h}^{2}=\sum_{i=1}^{n_{h}} \frac{(I_{h,i}-\overline{I}_{h})}{(n_{h}-1)}$ | Sample variance in the $h^{th}$ stratum |
| $\overline{I}=\sum_{h=1}^{H} W_{h}\overline{I}_{h}$ | Estimate of the population mean per unit (i.e. stratified mean catch per tow) |
| $s^{2}(\overline{I})=\frac{1}{N^{2}}\sum_{h=1}^{H} N_{h}(N_{h}-n_{h})\frac{s_{h}^{2}}{n_{h}}$ | Estimate of the variance of the stratified mean |
| $\hat{I}=N\overline{I}$ | Estimate of the population total over the survey area |
