# Supplementary figures and images for: SimSurvey: An R package for comparing the design and analysis of surveys by simulating spatially-correlated populations
Source: PLoS One. 2020 May 11;15(5):e0232822. doi: 10.1371/journal.pone.0232822 (PMC7213729; doi:10.1371/journal.pone.0232822)

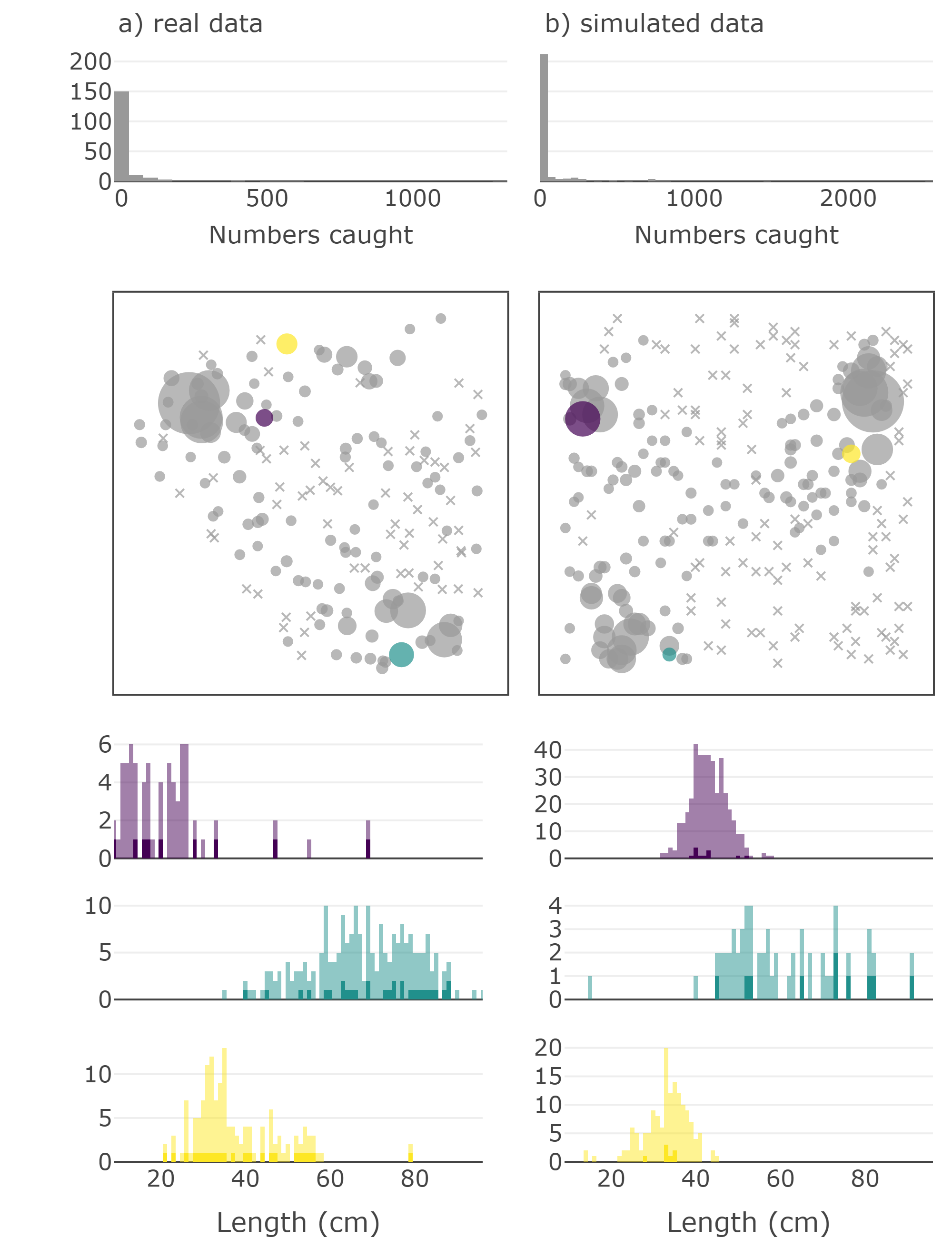

Supplement: S1 Fig — (PNG) [file pone.0232822.s006.png]
