# Supplementary material for: SimSurvey: An R package for comparing the design and analysis of surveys by simulating spatially-correlated populations
Source: PLoS One. 2020 May 11;15(5):e0232822. doi: 10.1371/journal.pone.0232822 (PMC7213729; doi:10.1371/journal.pone.0232822)
Supplement: Attachment — Submitted filename: rebuttal_letter.docx [file pone.0232822.s007.docx]

Daniel E. Duplisea, PhD
Fisheries and Oceans Canada
850 Route de la Mer
Mont‐Joli, Quebec
G5H 3Z4, Canada

2019-12-28

Dear Dr. Duplisea,

Thank you for considering a revision of the manuscript PONE-D-19-26904, **“SimSurvey: an R package to optimize the design and analysis of fisheries surveys by simulating spatially-correlated fish stocks”** by Paul M. Regular, Gregory J. Robertson, Keith P. Lewis, Jonathan Babyn, Brian Healey and Fran Mowbray. In accordance to one of the reviewers’ suggestions, the paper has been renamed **“SimSurvey: an R package for comparing the design and analysis of fisheries surveys by simulating spatially-correlated fish stocks”**.

We thank the Reviewers for their thoughtful and thorough comments which will clarify and improve the manuscript. We agree with many of the comments and have made every effort to incorporate these into the manuscript. Below are the reviewers’ comments with a brief accounting of our response to each concern or suggestion.

We submit this revised manuscript for your consideration and look forward to your decision.

Sincerely,

Paul Regular
Fisheries and Oceans Canada
Northwest Atlantic Fisheries Center
80 East White Hills, St. John’s, NL
A1C 5X1, Canada
E-mail: [Paul.Regular@dfo-mpo.gc.ca](mailto:Paul.Regular@dfo-mpo.gc.ca)
Phone: (709) 772-2067

Reviewer #1:

This paper describes the R package “SimSurvey” which is a package to test the design and analysis of fisheries independent surveys.

I found the paper very interesting and the package of great potential value and interest to many fishery scientists. However, I have several comments that I would like the authors to address first before I recommend acceptance of this paper. My main comments relate to:

1. Verify some of the equations and model description used in the model. I think that some of them lack description to fully understand what was done and few others are misleading, even incorrect. The detail can be found below but in general, they are linked to the problem of bias correction for log-normal distribution, re-scaling”, moving from abundance at age to abundance at length.
2. Add additional explanation to justify the choices. E.g. why population dynamics is modeled independently of the spatial distribution. Is this reasonable, limitation, etc.
3. Few suggestions (add-ons, renaming) to increase the generality of the paper (without requiring too much work)

*We hope we have addressed each of these main comments by a) clarifying several equations, b) adding more details and justifications, and c) modifying naming conventions and clarifying extensibility. See replies below for more details.*

Detailed comments:

Abstract:

What about the flexibility to include new sampling strategies (user defined one) or including new estimation approaches

*We have noted that both built-in and user-defined sampling strategies can be utilised.*

Introduction:

L43: quality of

*We have made the suggested change.*

It would be good to add few references here e.g. L50,

*We have added references as suggested.*

Method:

L69-73: I think it would be good to add the reference to the specific function name listed in Table2. This way, I would be more explicit and avoid possible confusion. For example, in my case, when I first read the section header “simulate abundance”, I was expecting to see the way you dealt with the spatial distribution of abundance.

*We have made the suggested change.*

L94&95&104: I just want to make sure that the bias correction has been applied to these two equations. If logN (or logZ) is normally distribution with mean mu and sigma, then the N (or Z) is lognormally distributed with mean exp(mu+sigma^2/2). Therefore, when you write “mean” in table 3 for R and Z, I hope that you modified in your actual equation for log(Z) to: log(Z) ~ normal(log(mean)-sigma^2/2, sigma) In other word, the mean(R) is not simply exp(meanR_log_scale). exp(meanR_log_scale) is the median of the distribution of R.

*Good catch. The answer to the question is no, bias correction was not applied, but nor was it needed, as all demographic parameters operated in log space. The issue is a matter of naming convention that we admittedly struggled with. When developing the functions, we vacillated on whether to use log_mean or mean as the argument name and we landed on mean as we believed this would be a more relatable value for users to supply. To be more explicit with regards to what value should be supplied, we have renamed the arguments to log_mean.*

L107: there is not enough description to understand how you converted the abundance at age to abundance at length. What were the choice of length bin groups? Every 5 cm, 10cm? Such information is also missing in the tables. Please add that. But then you have to calculate some cumulative distribution (until length l) for the length at age distribution (using the VB growth equation) and do some subtractions to be able to allocate the abundance at age to each length group. See equation A.1.14 in here for example. <https://www.nwfsc.noaa.gov/news/events/program_reviews/documents/C.2_Methot_Wetzel_SSTechnicalDescription.pdf>

*Another good catch as we did not provide enough information to replicate the approach. We have added more detail on the calculations behind the sim_vonB function; turns out our approach was similar to the one described in the exemplar the reviewer provided.*

“Simulate spatial distribution”:

I have a few comments on this section:

1. First of all, I think it is important to state that you assume that the population dynamic model and the spatial distribution is independent i.e. you do not have a spatially-explicit population dynamics model. And talk about what it means (is it realistic, limitation, etc).

*We have prefaced this section with the caveat that population and spatial dynamics are modeled as independent processes and we note the pros and cons of this approach. In short: the con is that the approach is a simplification of reality as it does not explicitly account for dynamics such as larval dispersal, spatial differences in growth and population connectivity; the pro is that this simplification limits the number of unknown parameters that need to be specified while facilitating the simulation of a sufficiently complex population for testing the efficacy of various survey designs.*

2. L161. I think it would be better to rename “depth” as “main covariate influencing the species distribution”. And “depth” happens to be one of them for many species, but there are cases when this is not the case.

*A good point, this variable could be used for any habitat gradient over which stratification is applied. However, we retain the term “depth” for three reasons: 1) it is an important covariate for many species (as the reviewer noted), 2) most surveys that we are aware of are depth-stratified (also noted by the reviewer below) and 3) it is a concise variable name (as opposed to something like “habitat_covar”). We therefore retained the “depth” naming as it ought to satisfy most use cases. We now note directly in the text that any covariate could be supplied and used as ‘depth’*

3. L161. I think it would be good to say upfront that there are two ways of defining the spatial structure. Using the function in the package or user defined.

*Given a suggestion from Reviewer #2, we have restructured the paper to have two core sections: “Model structure” and “Using* ***SimSurvey****”. We have included a blanket statement under the “Model structure” section noting that users can circumvent specific components of the* ***SimSurvey*** *framework and supply their own objects, including spatial structure of the survey grid.*

4. L161: I think another possibility is to generate a random field by using the package “Randomfields” for example. This way you can generate a map where “depth” is patchily distributed (maybe more like an island type case study)

*Good idea! We actually pursued this idea in an earlier iteration of make_grid, however, we abandoned the option because it was difficult to automate because the random field sometimes created small strata (one cell) or strata that were split in space. We would certainly welcome more development integrating other packages with* ***SimSurvey***

5. L163-164: please add more description about the division or strata. How can we set it up and what do you specifically mean by division and strata? Is one nested within the other, or not necessarily? Your examples are based on Atlantic Ocean and people in other regions might not be familiar with how these divisions are created.

*We have added more detail on the structure of the divisions and strata. In short, divisions are pre-defined areas, generally within which a fisheries management framework is applied. Age-length keys are specified at the level of the division, so for populations spanning large habitat gradients (in temperature for example) in which age-length relationships change along that gradient, multiple divisions can be supplied. Strata, on the other hand, are defined by an important habitat gradient. As structured in* ***SimSurvey****, strata are nested in divisions.*

6. L163: Why only focus on “depth-based strata”? I think it would be good to allow the user to choose their own stratification approach. It could be depth based as you did (which happens most often in surveys) but it could technically be any other thing (user supplied). This allows more flexibility.

*We agree, see reply to point 2. above, and note that any habitat variable could be used for stratification.*

7. L167: the equation is misleading and I am not sure it is right based on what is written in the text. You mentioned later on L178: that you “re-scaled” so that the total number of fish in a specific year and age across space is equal to the single number from the population dynamics model. If so, the re-scaling should be done in the identify (natural) scale, not in log scale. In log scale, even if ,, sums to zero across space, the sum in the identity scale won’t match. This is often refer to as “bias correction” for the log normal models. And you should ideally show how the rescaling was done in terms of the equations too.

*Right, the equation presented was not an accurate reflection of the calculations. We have revised the equation to explicitly show how the values were normalized to sum to 1.*

8. L167: this “depth preference” function is very simplistic and gives only very “smooth” symmetric distribution. More often, fish have a skewed depth preference: often right-skewed.

*True. Some users may find this parameterization insufficient for their species and we hope they will implement their own closure to use in the sim_distribution function to better simulate the effect. In addition to our blanket statement under the “Model structure” section, we have added a more specific statement under the “Using* ***SimSurvey****” section stating that alternate formulations can be used by supplying alternate closures to the core functions.*

9. L173-174: I think it might be worth adding, in simpler terms, the meaning of the spatial smoothing and scaling parameters.

*We have prefaced that sentence with “The rate at which point-to-point spatial correlation decays with distance is controlled by…”.*

10. L178: it is another question of scaling. How did you exactly do the scaling? In the identity scale? By dividing my the sum of the effects? I am asking this because depending on how you did the rescaling, your correlation structure in space and age might have been affected and is not the same as the one specified in L172. Did you verify that?

*See reply to point 7. above*

Table 4. “group_ages”. Ok but how is the variance controlled for the other age classes?

*“Variance” was a poor word choice. We have replaced it with “noise” as it is the simulated noise that we fix across multiple age groups.*

L189: “user supplied”. This is a good feature. However, I think it is important to mention here that user have to make sure that they use the correct projection method to ensure that each grid is of the same dimension.

*We have made the suggested change.*

L216: reference to figure 3?

*We have made the suggested change.*

L221: there is not “group_years” argument in Table 4.

*We have added it to the table.*

L237: “this function”. I think it would be better to replace “this function” with “sim_distribution” as you do not mention the word “sim_distribution” in the sentence above this.

*We have made the suggested change.*

L158-251: In general, I think re-organising this section using sub-section headers could be useful. Just to guide the readers

*This is an excellent suggestion. By following a suggestion by Reviewer #2 to re-organize the paper into two core sections we have added more headers to help guide the readers.*

L254: you say that sampling is stratified random but SRS is also an option based on Table5. Please correct.

*We have made the correction.*

L257: what does this mean? Does this control the number of set but how is this calculated?

*We have clarified how number of sets per strata is calculated.*

L257: I do not see how you control for the total number of set in the survey? How do you control it?

*We have clarified how number of sets per strata is calculated; it is done within the sim_survey function using the set_den argument, with further adjustment possible with the min_sets argument.*

L261: I think you should mention here that you can also force the sample size (as seen on table 5). Moreover, in table 5, it would be good to set-up a “ages_min” for the minimum number of ages to sample […]” so that it gives the ability to fix the sample size if needed by writing the same value for “ages_min” and “ages_cap”.

*We are not sure what the reviewer would like to have implemented here. Is the suggestion to impose a minimum number of ages to collect across all length groups? In practice, an ages_min constraint is already applied for length bins where the total number of fish caught is lower than ages_cap. A specific ages_min constraint cannot really be applied, as if there are too few fish of a certain size caught in any one survey, there is no means of getting more fish of that size.*

L261: How are you making sure that the number of sampled fish for that specific cell, age and year won’t be above the total number of fish in that cell, age, and year? The probability value could be close to one and if you fish in a few a time, then you are at risk. Especially because your population dynamics model is not spatially explicit and is completely independent of the distribution function itself i.e. you can technically fish out all the fish in an area but it will be populated back the year after the way you implemented in this study… Maybe you need to put a condition (or just a note) for general users to make sure that this probability value is much below 1?

*The sampling is implemented such that the number of fish sampled in a cell cannot exceed the number of fish in a cell because the population is split across sets in cases where more than one set is conducted in a cell. We have added this missing detail to our manuscript. We also added a note that the survey is assumed to have no impact on the population from one year to the next, and users planning to conduct surveys at levels that will affect the overall population will need to use caution in interpreting their results.*

L267: I recommend to clarify something here. 1. Depending on the number of fish caught? What do you mean? What is the rule you used? 2. The way you coded, sample by age is first decided, then the corresponding length is calculated, then age-subsample is determined. In reality, length sample is taken in the field, then age sub-samples are taken. While similar, I do not think it always equal. Especially, when you start including some correlation structure in the sampling. By the way, did you consider including some correlation structure in the sampling process to make more realistic?

*Our wording “depending on the number of fish caught” was unfortunately vague and, in hindsight, is not adding anything to the paper. Therefore, we have removed the statement to minimize confusion. We have also clarified the sub-sampling sequence. Finally, we have yet to consider including correlation structure in the sampling process as we went about imposing correlation via the spatial correlation of age groups (i.e. age-specific clustering tends to result in sets with high intraclass correlation). Correlation in the sampling procedure could be a future development for* ***SimSurvey****, but we feel at this time the package is sufficiently complex and we did not want to overwhelm users with too many options.*

L275 Table 1 on should be table 5

*We have changed the page numbers accordingly.*

L275: Table5: “age_sammpling” should be “age_sampling”

*We have made the suggested change.*

L275: “min_sets” you have not described it yet and what is it? You have sample from all cells? If no, this is not realistic.

*We have clarified the meaning and utility of the min_sets argument (i.e. a small strata may be allocated only one set under a low set density scenario; this argument overrides the allocation and imposes the min_sets if it is greater than the allocation).*

L279: Table5 not Table1?

*We have changed the numbers accordingly.*

L285-286: Could you be more specific on how custom closures can be supplied and where?

*We have included an example in the second paragraph of the “Using* ***SimSurvey****” section that we hope will clarify how a user can supply a custom closure.*

L306: how are these catchability corrected abundance matrices calculated? It is important to write this information somewhere (or write “please refer to the section “Stratified analysis” for further information on the calculation of abundance indices”) or something alike and Appendix S3.

*We have clarified how this was calculated.*

L336: I think it would be good to say that other methods exist and people can use it in this package (maybe)?

*Good point, however, we think this is covered by referencing a paper that describes a geostatistical R package and we also note that other options can be used under the “Research opportunities” section.*

L421: color gradient. Even though it is obvious it might be good to say green to purple gradient.

*We have made the suggested change.*

L427: instead of “sampling protocol”, I think it would be more meaning full to say the maximum number of length samples.

*We have made the suggested change.*

L452: say that the color ramps from yellow to purple

*We have made the suggested change.*

S1 appendix: missing figure in S1

*We have included the figure*

Reviewer #2:

This manuscript describes an R package called SimSurvey. The package includes a set of functions for simulating point-based fisheries survey designs, e.g. bottom trawl surveys, for estimating abundance indices. It focuses on number of stations and number of fish sampled ignores other constraints such as distance between stations and day-time duration which impose strong constraints on real surveys. The functions included allow the user to first simulate age-and length structured population dynamics, distribute individuals randomly in space (assuming a certain correlation structure), carry out a survey and finally calculate abundance indices from the simulated data. The package will likely be of interest to researchers wanting to explore the precision achievable with different survey designs. My comments regarding the package and the presentation are summarised below.

1. Optimization

The title announces a package for optimizing survey designs. As far as I can see the package does not allow survey design optimization, neither in terms of defining survey strata nor in terms of number of stations per stratum. The strata are defined by the user. The only option available for the number of stations is proportional to stratum surface; the user sets the minimum number of stations taken in the smallest stratum. It would be useful to be able to specify the total number of stations and test different allocation schemes, such as proportional to surface area (implemented), equal number per stratum, Neyman allocation (accounting for surface area and abundance variability), etc. Please consider revising the title (e.g. “compare” and instead of “optimize)” and spell out the available sampling design options.

*We agree with the Reviewer and retitled the ms “SimSurvey: an R package for comparing the design and analysis of fisheries surveys by simulating spatially-correlated fish stocks”. Currently the package provides options for stratified random sampling proportional to surface area, but other survey designs could added in future versions of* ***SimSurvey****.*

2. Manuscript structure

The manuscript might be easier to follow if the manuscript was restructured: 1) Model description, 2) Using SimSurvey. The later section would then group all example code which could again be subdivided into running simulations and exploring results (plot functions).

*This is an excellent suggestion! We have re-structured our manuscript accordingly and feel that this structure will be much easier for a reader to follow.*

3. Parameterisation To use SimSurvey for a real world problem realistic parameter values are needed. The package comes with default values chosen for a particular case study. However, no mention is made in the manuscript how to choose appropriate values for the many model parameters to tailor the simulations to a population of interest. I suggest the authors add a section on parameterisation and a table summarizing all parameters with a column specifying how to parameterize. For example, parameters for population dynamics and growth could probably be taken from the literature (or a stock assessment report). However this is not possible for the parameters of the spatial distribution function sim_distribution() such as correlation between ages etc. Ideally the package would include a fitting function for estimating these parameters from actual data. These input data could come from a pilot survey and include location (lat, long) and numbers by length/age.

*This is yet another very helpful suggestion and, now that we have included a Parameterisation section, it is easy to see the value of such a section to prospective users of the package. Instead of a table that summarizes how to specify the parameters, we wrote an entire section that outlines recommended steps for setting up a their own simulation. We started drafting a table, however, it quickly became apparent that it would be too big and cumbersome and somewhat redundant with the core tables that describe the arguments and parameters. We hope that the narrative/outline we included will serve the practical purpose the reviewer had in mind.*

Minor issues - line 93: I assume there is an age plus but this needs to be mentioned. Also, please specify how you set the initial numbers for plus group ().

*We have now noted in the manuscript that a plus group is not modeled explicitly as the number of ages can easily be extended to include groups with zero fish. This choice simplifies the simulation, including the setting of initial numbers which is done via exponential decay. Further, the lack of a plus group is inconsequential for survey based estimates of abundance at age.*

- line 121: I don’t understand the explanation of a closure. What do you mean by “return functions”? Do you mean it returns an object with different attributes?

*We have improved our explanation of a closure at the beginning of the Using* ***SimSurvey*** *section*

- line 125: the number of right and left brackets is unbalanced, please check

*We have provided an improved description of a closure; the logic behind this line of code should be clearer now*

- line 126 “This structure was chosen to avoid the repeated specifications of ages and years”. As far as I can see the example code only specifies years, not ages.

*Again, we hope that our improved description of a closure will clarify what we mean by this.*

- line 227 Please explain what a pipe is and how it is used. In the example I understand that the output of sim_abundance( ) is provided to (piped) into sim_distribution(). I am unclear what the object b contains. Is it the result of sim_distribution()?

*We have clarified how a pipe works, noting that it forwards values from one function call to the next function call, and we now state that the output from the two examples provided (nested approach vs. pipe approach) are functionally the same though the approach is slightly different.*

- There is no table 1, please revise table numbering.

*We have made the suggested change.*
