# Supplementary material for: SimSurvey: An R package for comparing the design and analysis of surveys by simulating spatially-correlated populations
Source: PLoS One. 2020 May 11;15(5):e0232822. doi: 10.1371/journal.pone.0232822 (PMC7213729; doi:10.1371/journal.pone.0232822)
Supplement: Attachment — Submitted filename: response_letter.docx [file pone.0232822.s008.docx]

Daniel E. Duplisea, PhD
Fisheries and Oceans Canada
850 Route de la Mer
Mont‐Joli, Quebec
G5H 3Z4, Canada

2020-02-19

Dear Dr. Duplisea,

Thank you for considering another revision of manuscript PONE-D-19-26904R1, **“SimSurvey: an R package for comparing the design and analysis of fisheries surveys by simulating spatially-correlated fish stocks”** by Paul M. Regular, Gregory J. Robertson, Keith P. Lewis, Jonathan Babyn, Brian Healey and Fran Mowbray. We are also grateful for your detailed suggestions and we have made every effort to do justice to the changes you recommend. Most importantly, we hope we have added sufficient content to the core of the paper to alleviate concerns that the manuscript is simply a software manual and further elevate the paper to be a suitable primary scientific publication. Though the how-to approach remains, we now see that describing some of the case study results in the core of the manuscript makes it more interesting and it adds another tangible reason for prospective users to ask survey design questions relevant to the their study system by learning how to use the package. Please see below for more details on the changes we made in response to your suggestions.

We submit this revised manuscript for your consideration and look forward to your decision.

Sincerely,

Paul Regular
Fisheries and Oceans Canada
Northwest Atlantic Fisheries Center
80 East White Hills, St. John’s, NL
A1C 5X1, Canada
E-mail: [Paul.Regular@dfo-mpo.gc.ca](mailto:Paul.Regular@dfo-mpo.gc.ca)
Phone: (709) 772-2067

Additional Editor Comments:

Dear Paul, you have made lots of excellent changes that I think help with the use of the package and responded to the many specific comments of reviewers which has no doubt corrected many technical issues and clarifications. The one thing I would say that you have not really done is get at the deeper research merits of this work beyond introducing a new piece of software. I think paper, as it stands, lacks a larger context and content which is important for the primary publication. My diagnosis for why this is is that the manuscript does not conform very well to more typical scientific reports (Intro, M&M, Result, Discussion) which can make it difficult for readers to find the larger scientific merits of the work. It is useful of course for those who already understand the merits of this kind of work but this work is for primary publication and it needs to appeal more to the former than the latter group. There is a very “how-to” feel to it (e.g. line 64 “In this section”) which I think detracts from getting at the larger purpose of the work.

I would really like you to address this issue of moving it from a software manual to a primary scientific publication. I do not think it should involve that much work but there will be some restructuring of sections as well as places to put in content and bring out conclusions. Here are my suggestions for this:

Try to follow a more traditional paper structure. This will help readers and it likely will also make it clearer for you on how you can inject content into the paper to move it beyond the software manual approach:

Introduction:

- You need to talk about wider issues and examples. e.g. examples of when poor survey design meant that scientific questions could not be properly addressed when good survey design meant they were. Examples of when good survey design allowed researchers to address needs that were unanticipated at the time it was designed. i.e. you need to build a better case (not just cost) of why survey design is really important and it is most powerful to do this with examples. You should try to bring in ideas related to ecosystem and climate changes and being able to track communities. Perhaps bring in species at risk ideas and tracking decline, you could bring in ideas related to MSC certification. These are just examples of specifics but you get the idea: the Introduction needs to have more general information outlining in both a broad and specific sense why we should be concerned about this.
- Keep in mind an educated reader who may not be in fisheries but is interested in why anyone should care about this or could, for example, be interested in surveying say caribou or songbirds or something outside of fisheries but where many of the motivation and concepts may be similar and they are doing a more general literature search before designing their own survey.

*Thank you for thinking beyond a fisheries biologist audience, as this is the audience we have been targeting from the onset. In hindsight we see that was short-sighted so we have modified our introduction, as suggested, to pitch the concept to a broader audience. The reason we did not think beyond fisheries biology is because we tend to have access to extensive age-based data while others (e.g. songbird and caribou biologists) do not. However, there are certainly other marine or terrestrial situations that could use our approach, the general framework we now present should be useful to adapt our work to systems we have not even considered.*

*Specifically, we have added an additional paragraph at the start of the paper discussing the importance of good survey design at a very general level. We also have removed references to fish and fisheries from some places in the abstract, to show our work can easily be generalized to other systems.*

Methods:

- This will start at your “Model Structure” section. You should put a higher level heading just before that called Methods.
- I can see that it is hard to separate your Methods from Results. Your results are really in the section “Using SimSurvey” I would not be opposed to putting this as a separate Results section but I leave it to you to decide. Essentially what you have is a case study as an example of how it works and therefore specific results are less interesting than how you got there with the package. You might title it something like “Results: running a SimSurvey simulation”. This section also has a lot of content without a lot of explanation. for example, you have several large and complicated figures in a row. You should discuss not only what figures are meant to offer in the package but also what they mean. So for example on line 473 you refer to three figures (5,6,7) but you offer little interpretation of those figures just why you can make them which is another example of the limitations of the how-to manual approach. So you need to think of it as a case study and help someone decide the implications of their survey design.

*We really struggled to accommodate traditional Methods and Results sections because all of our re-structuring attempts defied the definitions of these sections. To maintain a logical flow that describes the how and why to use the package, we landed on a mix of methods and case study results/discussion through the body of the paper. We first describe the “Model structure”, then the “Core functions”, and then we describe and discuss the case study results in the “Interpretation” section.*

*The new “Interpretation” section is essentially a shortened version of the appendix on the case study, however, this iteration of the manuscript includes one new result. In the process of revising the paper and reading more literature, we stumbled upon a potential design-based solution to the bias introduced by the division-level age-length key (default approach). The design-based fix was easily implemented using the package and, after running the necessary simulations, we discovered that the resultant estimates appear to be unbiased. We feel this is an interesting and useful addition to the paper as it shows that the package can be used to identify issues and explore solutions.*

*We should also note that we have moved the “Parameterisation” section to an appendix. This was the section where we provide some guidance on how to modify default settings to suit specific needs. Following the addition of the “Interpretation” section, it became clear that this section no longer fits with the logical flow of the paper.*

*Overall, we feel that the structural changes we have made to the paper have minimized the how-to content, and the increased focus on the case study should bring more meaning to the complex figures shown through the paper and highlight the real-world implications of the package.*

*Below we discuss a number of our failed attempts to place the content into Methods and Results sections:*

*1) We tried to start the methods section with “Model Structure”, as suggested, and shunt the figures to a “Results” section; this, however, broke the flow and left a gap in the methods section which held demonstrations of the core simulation functions but not the plotting functions.*

*2) We tried to keep the methods section as is and describe some of the case study results in the results section, however it seemed awkward to describe and discuss a solution to the age-length key problem in a results section.*

*3) Earlier iterations of the paper were focused entirely on the case study, the format was traditional and the package was a side-note in the methods section. It became apparent, however, that the package was more interesting and generally applicable than the case study results. We then began re-working the paper into a how-to format and, while moving away from a conventional format, we looked to several papers as a guide on how to document a R package (e.g.* [*https://journals.plos.org/plosone/article?id=10.1371/journal.pone.0092725*](https://journals.plos.org/plosone/article?id=10.1371/journal.pone.0092725)*).*

Results:

- see comment above

*Please see reply to Methods comment.*

Discussion:

- This starts just before “Research Opportunities”. You can keep these sections but there should be more preamble before jumping right into research opportunities. I suggest a general paragraph(s) that segue into your subsections of “research opportunities”, “future research”. In the Discussion you should address again some of the broader issues from the Introduction, e.g. how could spatial (depth) distribution changes anticipated under climate change for some population be tackled by survey design exploration now so that we can continue to track these changing populations 20 years down the road and do not lose the signal. How can this software help with that?

*In contrast to a Methods or Results section, a Discussion section was easier to accommodate and we have provided one. Here we lean on the case study results to reiterate the importance of planning a survey or testing an existing survey. We then use this preamble to segue into the “Research opportunities” section as suggested.*

- I think something that can be useful for readers is to outline the steps in a thought process a researcher might undertake when setting up a survey (perhaps a separate subsection) and then how one might go about a SimSurvey run for this. It also gives you a good opportunity to discuss your multispecies ideas:
  - What is your current problem/needs
  - What are the constraints (HR and $ perspective)
  - What are the constraints from a biological, physical perspective
  - Resources available
  - What are your anticipated future needs (i.e. if climate change is going to make cod go deeper, will you be able to track it in 20 years?)
  - How could you consider some or all of these with SimSurvey

*As much as we would like to address each of these points and work towards a survey design handbook, it would be hard to be as cohesive and comprehensive as Cochran (1977; “Sampling techniques”) or Sutherland (2006; “Ecological census techniques: a handbook”). Moreover, we have yet to implement features that could help users assess some of these trade-offs (i.e. cost-benefit analysis). We are hopeful that these are questions that future versions of SimSurvey may be able to help address as more features are added to elevate it from a purely statistical toolbox to something that also considers the operational constraints of delivering a survey.*

- Your “Assumptions” section should come down into the Discussion

*Agreed, we have made the suggested change.*

- “Future Directions” should say something about randomfields re: Reviewer 1. Even if you just outline that you have considered it. You might also try to say something about optimisation of design which Reviewer 2 mentioned.

*Good point. We have noted in the “sim_distribution” section that a custom closure can be created that uses randomfields to simulate spatial noise. We have also noted in the “Future directions” section that we hope to implement alternatives to random or stratified random designs to allow for more comprehensive evaluations of various designs.*
