# Supplementary material for: SimSurvey: An R package for comparing the design and analysis of surveys by simulating spatially-correlated populations
Source: PLoS One. 2020 May 11;15(5):e0232822. doi: 10.1371/journal.pone.0232822 (PMC7213729; doi:10.1371/journal.pone.0232822)
Supplement: Attachment — Submitted filename: response_letter.docx [file pone.0232822.s009.docx]

Daniel E. Duplisea, PhD
Fisheries and Oceans Canada
850 Route de la Mer
Mont‐Joli, Quebec
G5H 3Z4, Canada

2020-04-16

Dear Dr. Duplisea,

Thank you for considering another revision of manuscript PONE-D-19-26904R2, **“SimSurvey: an R package for comparing the design and analysis of fisheries surveys by simulating spatially-correlated fish stocks”** by Paul M. Regular, Gregory J. Robertson, Keith P. Lewis, Jonathan Babyn, Brian Healey and Fran Mowbray. We have carefully considered the latest round of suggestions and below we outline how we have addressed each point.

We submit this revised manuscript for your consideration and look forward to your decision.

Sincerely,

Paul Regular
Fisheries and Oceans Canada
Northwest Atlantic Fisheries Center
80 East White Hills, St. John’s, NL
A1C 5X1, Canada
E-mail: [Paul.Regular@dfo-mpo.gc.ca](mailto:Paul.Regular@dfo-mpo.gc.ca)
Phone: (709) 772-2067

Reviewer #1: This is my second time reviewing this manuscript and the authors did a great job addressing people’s comments. Great job for that. The manuscript is now much clearer and read well, I think.

*We thank the reviewer for the kind words.*

This time, I only have a few minor comments:
L19 : «there is a limited number of»

*We have removed the ‘the’ that should not have been in that sentence.*

L103: Maybe it would be a good idea to justify why you are converting to abundance at length.

*Agreed, we have started a new paragraph regarding abundance at length which begins: “In practice, abundance at age is often inferred from length data as it is easier to collect. Abundance at length is therefore simulated from abundance at age using the original von Bertalanffy growth curve”*

L240-242: I think it could be a good idea to show how to exactly do this (maybe obvious to advanced people but not to the general audience). This could be a simple reference to an example on the github repo.

*Indeed, this is a helpful idea and we have now created a short vignette on creating closures and this can be accessed via the pkgdown site (*[*https://paulregular.github.io/SimSurvey/articles/custom_closures.html*](https://paulregular.github.io/SimSurvey/articles/custom_closures.html)*). A link to this vignette has been added to the paper.*

Table 2: If bias correction was not implemented, I would suggest to change the labeling in the table here and elsewhere (if needed) and replace the “mean” by “median” (if the variable is transformed back to the original scale). If not, I would make clear that you talk about mean in log scale.

*Good catch, we have added log after mean to ensure that readers know the functions are operating in log scale.*

L326: I did not see how one can use INLA or RandomFields in Appendix S3. This is a similar comment as the one for line 240-242.

*We have added the following in parentheses: see the code behind sim_ays_covar_sped [*[*https://github.com/PaulRegular/SimSurvey/blob/master/R/sim_dist_spde.R*](https://github.com/PaulRegular/SimSurvey/blob/master/R/sim_dist_spde.R)*] for an example of how the sim_ays_covar closure was modified to apply a Stochastic Partial Differential Equation approach using the INLA package.*

L481-490: the processing time is surprisingly long… it is not an issue in itself but this might refrain some people to using this package… I think some code optimization could help (e.g. vectorize, use matrix operation as much as possible, etc)

*We have stretched our programming skills to the limit to reduce the processing time as much as possible. Of course, said programming skills are largely self-taught, so there is surely room for improvement. While developing the package, we repeatedly profiled our code and vectorized as much as we could, leaning heavily on the data.table package. We have also applied parallel processing in places in an attempt to speed up the code. In other parts of the package, we learned that there is little we can do to reduce the processing time (e.g. Cholesky decomposition is a factor limiting the speed of the sim_distribution function). Another step to further optimize the code would be to translate some of the core functions to C++, however this is on the wish list and well outside the scope of the project at this point in time. We are hopeful that we have produced enough here to catalyze collaborations that help extend and optimize the package.*
